# Supplementary material for: Empirical methods for controlling false positives and estimating confidence in ChIP-Seq peaks
Source: BMC Bioinformatics. 2008 Dec 5;9:523. doi: 10.1186/1471-2105-9-523 (PMC2628906; doi:10.1186/1471-2105-9-523)
Supplement: Additional File 1 — A variety of html documents from the USeq web site detailing the available applications, their best usage, output file type descriptions, command line menus, etc. [file 1471-2105-9-523-S1.zip › USeqUserGuides/JavaDoc.zip!/JavaDoc/gata/aligner/package-summary.html]

gata.aligner


|  |  |  |
| --- | --- | --- |
| **Overview** \| **Package** \| Class \| **Use** \| **Tree** \| **Deprecated** \| **Index** \| **Help** | |  |
| **PREV PACKAGE**   **NEXT PACKAGE** | **FRAMES**    **NO FRAMES**     **All Classes** |  |


---

## Package gata.aligner

For aligning and post processing DNA sequences using BLAST for subsequent import into GATAPlotter.

**See:**
  
          **Description**

| **Class Summary** | |
| --- | --- |
| **AlignerInputForm** |  |
| **AlignerInputPanel** |  |
| **AlignerPreferences** |  |
| **AlignerThread** |  |
| **Alignment** |  |
| **AlignParams** |  |
| **Blast** |  |
| **BrowseButtonAction** |  |
| **DelmeAligner** |  |
| **FileFocusListener** |  |
| **GATAligner** |  |
| **LocalAlignment** |  |
| **Seqs** |  |

## Package gata.aligner Description

For aligning and post processing DNA sequences using BLAST for subsequent import into GATAPlotter.

---


|  |  |  |
| --- | --- | --- |
| **Overview** \| **Package** \| Class \| **Use** \| **Tree** \| **Deprecated** \| **Index** \| **Help** | |  |
| **PREV PACKAGE**   **NEXT PACKAGE** | **FRAMES**    **NO FRAMES**     **All Classes** |  |


---
